# Long-term predictors of developmental outcome and disease burden in *SCN1A*-positive Dravet syndrome

**DOI:** 10.1093/braincomms/fcae004

**Published:** 2024-01-09

**Authors:** Tony Feng, Phoebe Makiello, Benjamin Dunwoody, Felix Steckler, Joseph D Symonds, Sameer M Zuberi, Liam Dorris, Andreas Brunklaus

**Affiliations:** School of Health and Wellbeing, University of Glasgow, Clarice Pears Building, 90 Byres Road, Glasgow G12 8TB, UK; The Paediatric Neurosciences Research Group, Royal Hospital for Children, Office Block, Level 0, Zone 1, 1345 Govan Road, Glasgow G51 4TF, UK; The Paediatric Neurosciences Research Group, Royal Hospital for Children, Office Block, Level 0, Zone 1, 1345 Govan Road, Glasgow G51 4TF, UK; The Paediatric Neurosciences Research Group, Royal Hospital for Children, Office Block, Level 0, Zone 1, 1345 Govan Road, Glasgow G51 4TF, UK; School of Health and Wellbeing, University of Glasgow, Clarice Pears Building, 90 Byres Road, Glasgow G12 8TB, UK; The Paediatric Neurosciences Research Group, Royal Hospital for Children, Office Block, Level 0, Zone 1, 1345 Govan Road, Glasgow G51 4TF, UK; School of Health and Wellbeing, University of Glasgow, Clarice Pears Building, 90 Byres Road, Glasgow G12 8TB, UK; The Paediatric Neurosciences Research Group, Royal Hospital for Children, Office Block, Level 0, Zone 1, 1345 Govan Road, Glasgow G51 4TF, UK; School of Health and Wellbeing, University of Glasgow, Clarice Pears Building, 90 Byres Road, Glasgow G12 8TB, UK; The Paediatric Neurosciences Research Group, Royal Hospital for Children, Office Block, Level 0, Zone 1, 1345 Govan Road, Glasgow G51 4TF, UK; School of Health and Wellbeing, University of Glasgow, Clarice Pears Building, 90 Byres Road, Glasgow G12 8TB, UK; The Paediatric Neurosciences Research Group, Royal Hospital for Children, Office Block, Level 0, Zone 1, 1345 Govan Road, Glasgow G51 4TF, UK; School of Health and Wellbeing, University of Glasgow, Clarice Pears Building, 90 Byres Road, Glasgow G12 8TB, UK; The Paediatric Neurosciences Research Group, Royal Hospital for Children, Office Block, Level 0, Zone 1, 1345 Govan Road, Glasgow G51 4TF, UK

**Keywords:** *SCN1A*, Dravet syndrome, epilepsy, comorbidities, developmental outcome

## Abstract

Dravet syndrome is a severe infantile onset developmental and epileptic encephalopathy associated with mutations in the sodium channel alpha 1 subunit gene *SCN1A*. Prospective data on long-term developmental and clinical outcomes are limited; this study seeks to evaluate the clinical course of Dravet syndrome over a 10-year period and identify predictors of developmental outcome. *SCN1A* mutation-positive Dravet syndrome patients were prospectively followed up in the UK from 2010 to 2020. Caregivers completed structured questionnaires on clinical features and disease burden; the Epilepsy & Learning Disability Quality of Life Questionnaire, the Adaptive Behavioural Assessment System-3 and the Sleep Disturbance Scale for Children. Sixty-eight of 113 caregivers (60%) returned posted questionnaires. Developmental outcome worsened at follow-up (4.45 [SD 0.65], profound cognitive impairment) compared to baseline (2.9 [SD 1.1], moderate cognitive impairment, *P* < 0.001), whereas epilepsy severity appeared less severe at 10-year follow-up (*P* = 0.042). Comorbidities were more apparent at 10-year outcome including an increase in autistic features (77% [48/62] versus 30% [17/57], χ^2^ = 19.9, *P* < 0.001), behavioural problems (81% [46/57] versus 38% [23/60], χ^2^ = 14.1, *P* < 0.001) and motor/mobility problems (80% [51/64] versus 41% [24/59], χ^2^ = 16.9, *P* < 0.001). Subgroup analysis demonstrated a more significant rise in comorbidities in younger compared to older patients. Predictors of worse long-term developmental outcome included poorer baseline language ability (*P* < 0.001), more severe baseline epilepsy severity (*P* = 0.003) and a worse *SCN1A* genetic score (*P* = 0.027). Sudden unexpected death in epilepsy had not been discussed with a medical professional in 35% (24/68) of participants. Over 90% of caregivers reported a negative impact on their own health and career opportunities. Our study identifies important predictors and potential biomarkers of developmental outcome in Dravet syndrome and emphasizes the significant caregiver burden of illness. The negative impact of epilepsy severity at baseline on long-term developmental outcomes highlights the importance of implementing early and focused therapies whilst the potential impact of newer anti-seizure medications requires further study.

## Teaser

Feng *et al*. report that poorer baseline language ability, more severe baseline epilepsy severity and a worse *SCN1A* genetic score were significant predictors of worse developmental outcome in Dravet syndrome at 10-year follow-up. The identified disease biomarkers emphasize the importance of implementing early and focused therapies in Dravet syndrome.

## Graphical Abstract

**Graphical Abstract:**
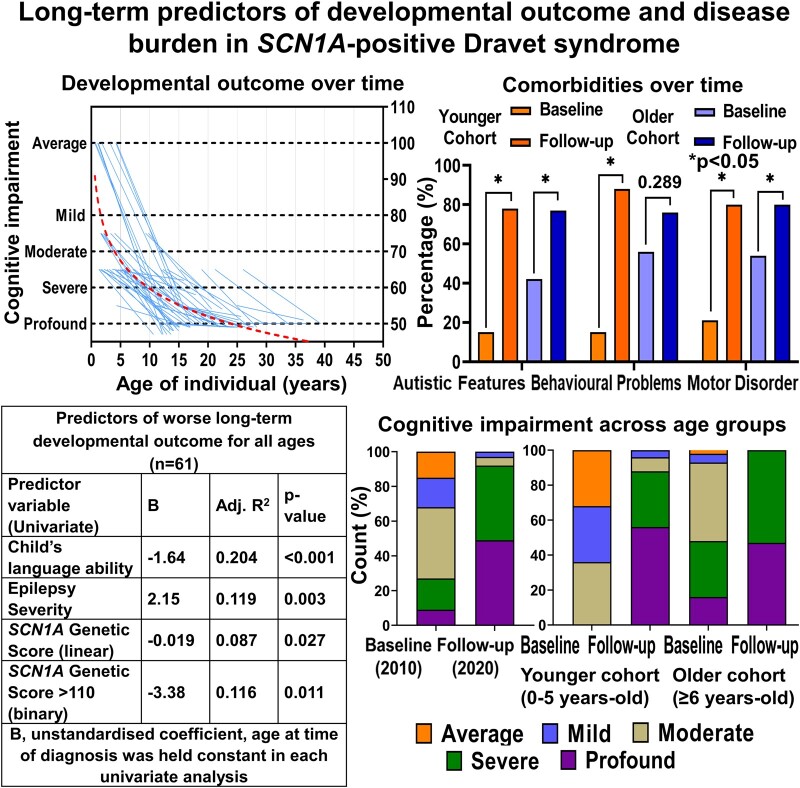

## Introduction

Dravet syndrome (DS) is one of the most common monogenic epilepsies, presenting as a developmental and epileptic encephalopathy resulting in cognitive, behavioural and motor impairments.^1,2^ DS is caused by loss-of-function (LoF) mutations in the *SCN1A* gene^3^ and children typically experience seizure exacerbation following sodium channel blocker (SCB) use. It has been suggested that early diagnosis combined with appropriate, focused therapy may improve long-term cognitive outcomes, however current evidence-based treatment does not appear to substantially alter the disease course.^4-7^ More recently, novel treatments have demonstrated better efficacy in seizure control, including cannabidiol, fenfluramine and stiripentol,^8-10^ and new *SCN1A* disease-modifying therapies targeting LoF variants are being developed.^11^

A cross-sectional study of mutation-positive DS individuals systematically examining prognostic, clinical and demographic features of DS identified independent predictors of poor developmental outcome, encouraging early counselling and syndrome-specific therapy.^12^ Whilst studies have demonstrated the significant disease impact in individuals with DS from child to adulthood,^13-15^ better understanding of long-term predictors of developmental outcome would aid counselling and therapeutic planning.

Here, we present the findings of a prospective longitudinal 10-year follow-up study with the objective of evaluating clinical and demographic features and to identify short- and long-term predictors of developmental outcome in *SCN1A* mutation-positive DS.

## Materials and methods

### Study design and participants

This is a 10-year follow-up to a 2010 study cohort of *SCN1A* mutation-positive DS individuals referred to the Epilepsy Genetics Service in Glasgow, between November 2005 and February 2010.^12^

Participants were asked to complete four postal questionnaires; a structured generic questionnaire on clinical features and disease burden, the Epilepsy & Learning Disability Quality of Life Questionnaire (ELDQOL),^16^ the Adaptive Behavioural Assessment System (ABAS-3) and the Sleep Disturbances Scale for Children (SDSC). Comorbidity features were documented by the clinician at baseline and via structured parent/caregiver questionnaire at follow-up. Detailed methods can be found in the original reports.^12,17^ Baseline study questionnaires were collected between 2009 and 2010, and all follow-up questionnaires were collected 10 years later between 2019 and 2020.

In the initial study, the developmental outcome was classified by clinicians with expertise in assessing developmental outcome using a Likert scale as 1 = average, 2 = mild cognitive impairment, 3 = moderate cognitive impairment, 4 = severe cognitive impairment and 5 = profound cognitive impairment. At follow-up, developmental outcome was based on ABAS-3, a caregiver completed questionnaire that makes a norm-referenced assessment of adaptive skills by assessing three major domains (conceptual, social and practical) across 11 skill areas, the aggregate of which is the General Adaptive Composite (GAC) score.^18^ For the purpose of comparison, a GAC score of 80–100 was defined as 1 = ‘average range’, 70–80 as 2 = ‘mild’, 60–70 as 3 = ‘moderate’, 50–60 as 4 = ‘severe’ and all scores < 50 as 5 = ‘profound’ cognitive impairment.

The SDSC is a caregiver-reported 26-item Likert scale questionnaire to screen for the presence of sleep difficulties and evaluate sleep profiles within the past six months.^19^ It combines six categories of sleep disorders: disorders of initiating and maintaining sleep (DIMS), sleep breathing disorders (SBD), disorders of arousal (DA), sleep–wake transition disorders (SWTD), disorders of excessive somnolence (DOES) and sleep hyperhidrosis (SH). Questionnaires were scored according to the SDSC score, generating a subcategory and total sleep score that could be classified as abnormal, borderline or normal.

To identify whether genetic information of DS individuals predicted adaptive skills at 10-year follow-up, we used the recently published *SCN1A* genetic score. The higher the score, the more deleterious the mutation (range: 0–207).^20^

Comorbidities and predictors of health-related quality of life of this cohort have been reported elsewhere.^21^

### Ethics statement

This study was approved by the Scotland A Research Ethics Committee (reference 08/MRE00/115), and informed consent was obtained from each study participant or their parent or guardian in the case of minors.

### Data analysis

Individuals with missing data were excluded from the relevant analyses. Three individuals at follow-up were identified as having non-DS phenotypes due to their average cognition on ABAS-3 testing and were excluded from the regression analysis. Linear regression models were used to predict developmental outcomes at baseline and follow-up. Baseline factors were tested as possible predictors in both the baseline and long-term follow-up model and included the following: gender, age at first seizure (months), presence and age at onset of different seizure types (Supplementary Table 1), epilepsy severity (as per ELDQOL), autistic features (yes/no), behavioural difficulties (yes/no), acquired motor disorder (yes/no), language ability (as per ELDQOL), EEG abnormalities in year 1 (yes/no), mutation type (truncating/missense), *SCN1A* genetic score and sodium channel blockers increasing seizure frequency (yes/no). Children were assessed at different ages that was identified as a potential confounder, hence the age at assessment was adjusted for and held constant throughout regression analysis. McNemar’s test was used to determine if there was a difference in phenotypic differences at follow-up and paired sample *t*-tests were used to assess differences across the follow-up period. DS individuals experience significant neurodevelopmental plateauing with emergence of behavioural and social difficulties in the first five years of life^17^ and subgroup analysis was performed to identify whether specific predictors could be identified by comparing those under or over six years old at age of baseline assessment. Statistical tests were two-tailed, and the alpha level used to determine significance was set to 0.05 (5%). Analysis was performed using SPSS version 26.0.

## Results

We contacted the clinicians of 141 participants that took part in the original study, of whom 140 responded. Ten individuals were lost to follow-up, seven individuals died (5.8%) and a further 10 developed non-DS *SCN1A*-related phenotypes (GEFS+, FS+ and MAE) that was not known at baseline in 2010 due to the young age of these individuals at the time. One hundred thirteen individuals who exhibited a DS phenotype and who had a positive *SCN1A* mutation were posted questionnaires, of whom 68 (60%) responded.^21^ The median age was seven years at baseline assessment (6 months to 42 years old, IQR = 4–15) and 17 (10 to 39 years old, IQR = 14–24) at follow-up. Sixty-two out of 141 (44%) were male in the initial study, compared to 36 out of 68 (53%) at follow-up (*P* = 0.7). A family history of febrile seizures or epilepsy was reported in 36 out of 141 cases (26%) in the initial study, compared to 17 out of 60 (28%) at follow-up (*P* = 0.2). For subgroup analysis according to age at baseline assessment (<6 years or ≥6 years), 28 individuals were in the younger group (median age 13 years at follow-up, IQR = 12–14) and 40 individuals were in the older group (median age 23 years at follow-up, IQR = 19–26). A total of 27 (40%) out of 68 had a missense mutation, and 41 (60%) had a protein truncating variant (PTV). Demographic and phenotypic cohort characteristics are detailed in Supplementary Table 1.

At follow-up, 7 out of 120 (5.8%) individuals with *SCN1A* positive DS were deceased. The majority were attributed to Sudden Unexpected Death in Epilepsy (SUDEP, 4/7) and the remaining three cases were due to status epilepticus, acute respiratory distress syndrome and one unknown cause.^21^ When asked whether SUDEP had been discussed with a medical professional, 24 of 68 carers (35%) indicated that this was not the case.

### Seizure progression

Carers of individuals with *SCN1A* positive DS reported epilepsy severity (as per ELDQOL) to be less severe at follow-up (1.86, SD 0.93) compared to their baseline assessment (1.63, SD 0.76, *t*(64) = 2.07, *P* = 0.042). This difference was greater for the older cohort (1.45, SD [baseline] 0.69 versus 1.76, SD 0.82 [follow-up], *t*[37] = 2.5, *P* = 0.016), whereas the younger cohort showed no significant change. Over two-thirds of patients (69%) reported medications exacerbating seizures across the lifespan (47/68). This included lamotrigine 49% (23/47), carbamazepine 23% (11/47) and phenytoin 15% (7/47; Supplementary Table 2).

### Developmental outcome: baseline versus follow-up

Overall, DS individuals had significantly worse developmental outcomes at follow-up (4.45, profound disability, SD 0.65) compared to their baseline (2.9, moderate disability, SD 1.1), *t*(60) = 10.66, *P* < 0.001 (Tables 1 and 2).

**Table 1:** Paired *t*-test comparing developmental outcome at baseline versus follow-up and split by age at baseline assessment (0–5 and ≥6 years old, total *n* = 61)

Ages (*N*)	Baseline mean (SD)	FU mean ± (SD)	Mean change ± (95% CI), *t*(df)	*P*-value
All ages (61)	2.9 (1.1)	4.45 (0.65)	1.55 (1.26 to 1.84), *t*(60) = 10.66	<0.001
Younger (25)	2.12 (0.83)	4.4 (0.82)	2.28 (1.84 to 2.71), *t*(24) = 10.74	<0.001
Older (36)	3.46 (0.92)	4.49 (0.51)	1.03 (0.73 to 1.32), *t*(35) = 7.1	<0.001

All paired comparisons were significant (*P* < 0.001); *N*, number; SD, standard deviation; CI, confidence interval; *t*, *t*-test; df, degrees of freedom.

**Table 2:** ABAS composite skill areas by group (*n* = 61)

Standard scores	Mean (SD)	Median (range)
GAC	51.15 (4.6)	49 (47–75)
Conceptual	54.4 (4.58)	54 (49–79)
Social	60.57 (6.67)	58 (54–84)
Practical	50.49 (3.07)	50 (48–63)

GAC, General Adaptive Composite.

**Figure 1:**
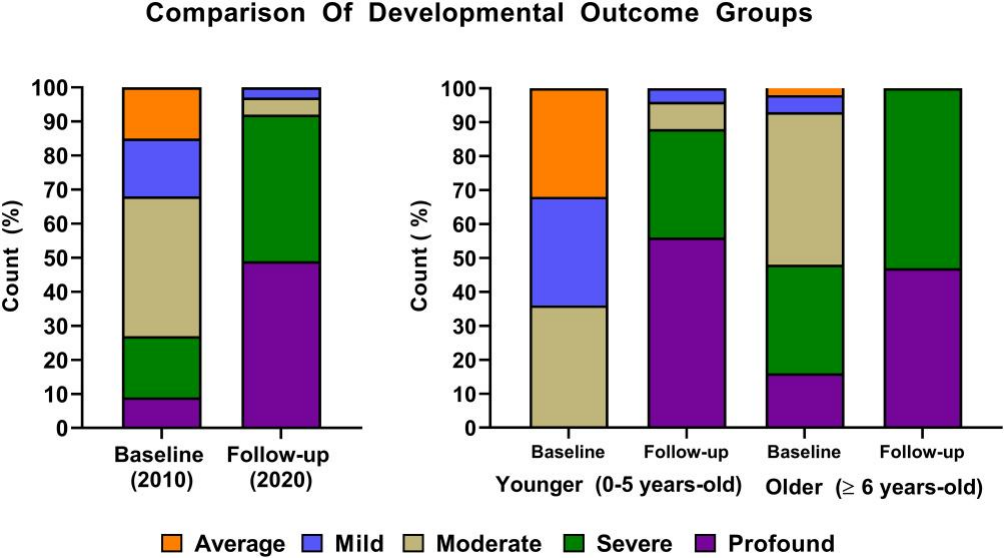
**Cross-sectional comparison of developmental outcome at baseline versus follow-up and split by age at baseline assessment (0–5 and ≥6 years old).** Developmental outcome was defined as ‘average range’, ‘mild’, ‘moderate’, ‘severe’ and ‘profound’ cognitive impairment.

**Figure 2:**
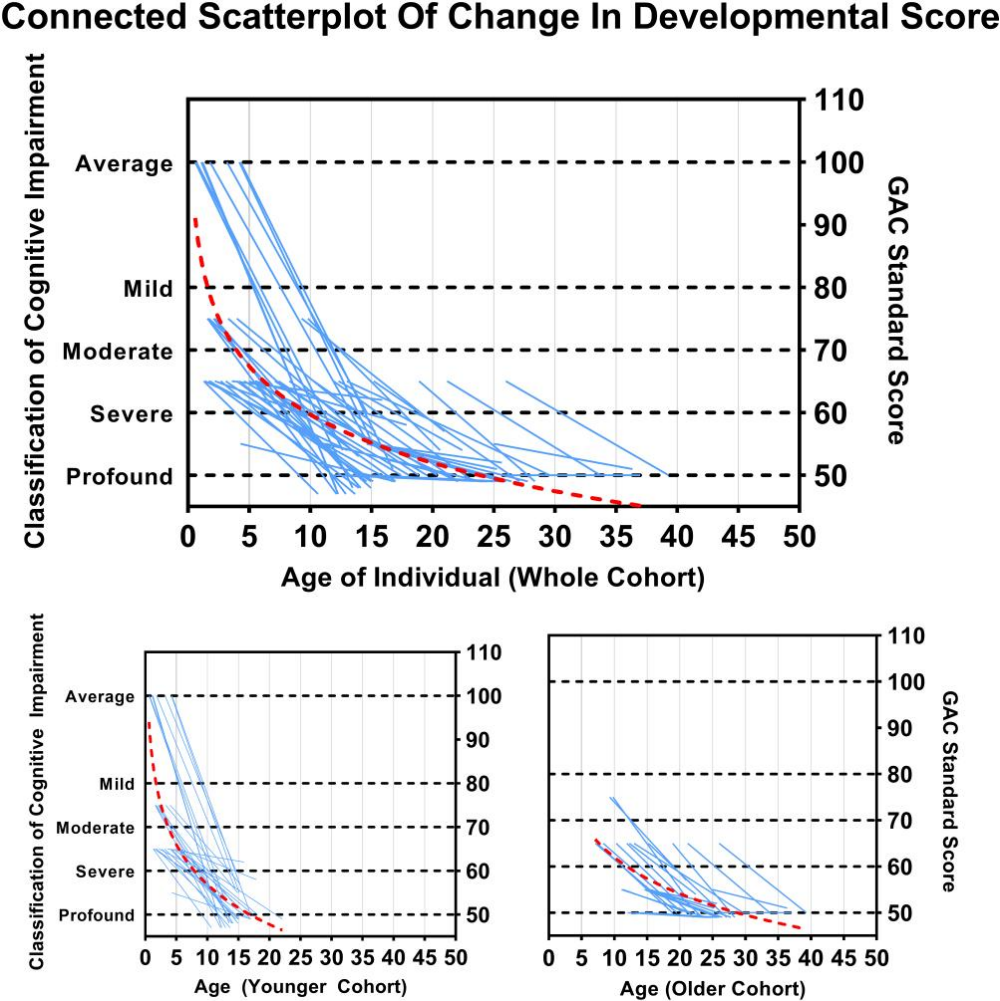
**Connected scatterplot of developmental outcome.** A General Adaptive Composite (GAC) score of 80–100 was defined as ‘average range’, 70–80 as ‘mild’, 60–70 as ‘moderate’, 50–60 as ‘severe’ and all scores < 50 as ‘profound’ cognitive impairment. Each line represents one individual’s decline in developmental score from the left *y*-axis (baseline developmental scores on a Likert scale) to the right *y*-axis (follow-up developmental scores as assessed by GAC standard score).

Subgroup analysis comparing the age groups showed that the younger group had a steeper decline in developmental outcomes compared to the older age group. For example, whilst none of the individuals in the younger group were considered to have severe or profound disability at baseline, this increased to 32% (8/25) and 56% (14/25), respectively, at follow-up (Figs 1 and 2).

### Comorbidities and disease burden

**Figure 3:**
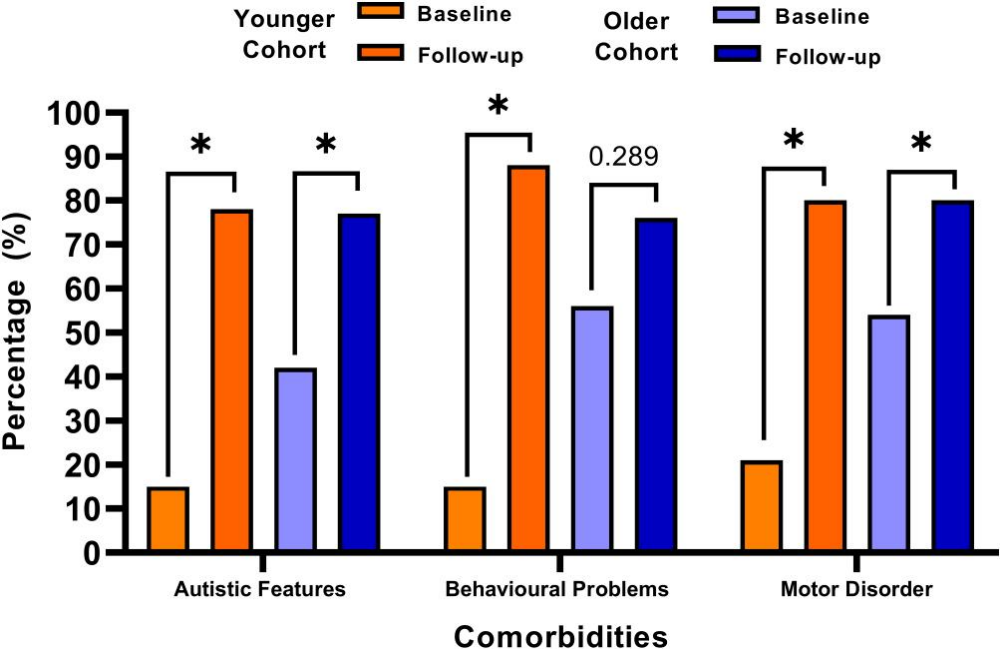
**Percentage of individuals with comorbidities observed at baseline versus follow-up, split by age at baseline assessment (0–5 and ≥6 years old).** *P < 0.05 (chi-squared test). Autistic features younger cohort baseline versus follow-up (χ^2^ = 14.1, *P* < 0.001). Autistic features older cohort baseline versus follow-up (χ^2^ = 4.92, *P* = 0.022). Behavioural problems younger cohort baseline versus follow-up (χ^2^ = 13.07, *P* < 0.001). Behavioural problems older cohort baseline versus follow-up (χ^2^ = 1.13, *P* = 0.289). Motor disorder younger cohort baseline versus follow-up (χ^2^ = 12.07, *P* = 0.001). Motor disorder older cohort baseline versus follow-up (χ^2^ = 4.1, *P* = 0.039).

Many comorbidities accrued across the 10-year follow-up period. Among these were an increase in autistic features at 77% (48/62) up from 30% (17/57), χ^2^(1) = 19.9, *P* < 0.001, behavioural problems at 81% (46/57) up from 38% (23/60), χ^2^(1) = 14.1, *P* < 0.001 and motor/mobility problems at 80% (51/64) up from 41% (24/59), χ^2^(1) = 16.9, *P* < 0.001. Subgroup analysis demonstrated a more significant rise in comorbidities in the younger group compared to the older group (Fig. 3, Table 3).

**Table 3:** Comparison of comorbidities at baseline and follow-up

	Baseline	Follow-up	Chi-square test	
Feature	Occurrence number/total (%)	Occurrence number/total (%)	χ^2^	*P*-value
Younger cohort				
Autistic features	4/26 (15%)	21/27 (78%)	14.1	<0.001
Behavioural problems	4/26 (15%)	21/24 (88%)	13.07	<0.001
Motor/mobility problems	5/24 (21%)	20/25 (80%)	12.07	0.001
Older cohort				
Autistic features	13/31 (42%)	27/35 (77%)	4.92	0.022
Behavioural problems	19/34 (56%)	25/33 (76%)	1.13	0.289
Motor/mobility problems	19/35 (54%)	31/39 (79%)	4.1	0.039

Forty-nine per cent (31/63) of individuals reported dental, 55% (35/64) eating problems and 18% (12/68) required a gastrostomy. Forty per cent (27/68) of individuals experienced fractures and 10% (7/68) reported to have a scoliosis (Table 4). In activities of daily living, individuals were fully dependent on carers in 66% (43/65), and partially independent in 34% (22/65) of cases. Caregiver’s health and job/career were negatively affected in 99% (65/66) and 91% (60/66) of cases, respectively. Whilst 72% of carers (47/65) reported access to respite care, only 52% (23/44) considered this to be sufficient. A summary of disease burden and analysis can be found in Table 4.

**Table 4:** Comorbidities and disease burden of *SCN1A* mutation-positive DS at follow-up (*n* = 68)

	All Ages (68)	Younger cohort (28)	Older cohort (40)	Chi-square/Fisher’s exact test	
	Occurrence number/total (%)	Occurrence number/total (%)	Occurrence number/total (%)	χ^2^	*P*-value
Comorbidities					
Autistic features	48/62 (77%)	21/27 (78%)	27/35 (77%)	0.004	0.953
Formal autistic spectrum disorder diagnosis	41/65 (63%)	20/26 (77%)	21/39 (54%)	3.567	0.059
Behavioural problems	46/57 (81%)	21/24 (88%)	25/33 (76%)	1.23	0.267
ADHD diagnosis	6/63 (10%)	2/25 (8%)	4/38 (11%)	0.112	0.738
Gastrointestinal					
Eating problems	35/64 (55%)	16/26 (62%)	19/38 (50%)	0.829	0.429
Requires gastrostomy/feeding tube	12/68 (18%)	7/28 (25%)	5/40 (13%)	1.771	0.183
Dental problems	31/63 (49%)	10/25 (40%)	21/38 (55%)	1.406	0.236
Mobility/orthopaedic					
Motor/mobility problems	51/64 (80%)	20/25 (80%)	31/39 (79%)	0.002	0.960
Requires walking aids	29/68 (43%)	8/28 (29%)	21/40 (53%)	5.127	0.077
Insoles/splints	25/68 (37%)	7/28 (25%)	18/40 (45%)	2.834	0.092
Wheelchair	19/68 (28%)	5/28 (18%)	14/40 (35%)	2.404	0.121
Experienced bone fractures	27/68 (40%)	9/28 (32%)	18/40 (45%)	1.137	0.286
Scoliosis	7/68 (10%)	2/28 (7%)	5/40 (13%)	0.512	0.474
Access to additional therapies					
Physiotherapies	34/68 (50%)	12/28 (43%)	22/40 (55%)	0.971	0.324
Occupational therapy	32/68 (47%)	14/28 (50%)	18/40 (45%)	0.165	0.684
Speech and language therapy	33/68 (49%)	18/28 (64%)	15/40 (38%)	4.731	0.030
Dietician	21/68 (31%)	8/28 (29%)	13/40 (33%)	0.119	0.730
Sleep and risk					
Sleep problems	45/67 (67%)	18/28 (64%)	27/39 (69%)	0.892	0.640
If yes, this has been treated with:					
Medication	23/45 (51%)	9/18 (50%)	14/27 (52%)	0.015	0.903
Sleep hygiene	7/45 (16%)	3/18 (17%)	4/27 (15%)	0.028	0.867
Untreated	18/45 (40%)	7/18 (39%)	11/27 (41%)	0.015	0.901
Treatment successful	13/25 (52%)	6/10 (60%)	7/15 (47%)	0.087	0.957
Parent sleeps in same room as child/adult	26/65 (40%)	11/28 (39%)	15/37 (41%)	0.01	0.919
Caregiver reports not having had a discussion about SUDEP	24/68 (35%)	10/28 (36%)	14/40 (35%)	0.752	0.687
Care and disease burden					
Child/adult lives with:					
Family	61/68 (90%)	28/28 (100%)	33/40 (85%)	-	-
Residential care	6/68 (9%)	0/28	6/40 (18%)	-	-
Both	1/68 (1%)	0/28	1/40 (3%)	-	-
Child/adult is:					
Partially independent	22/65 (34%)	8/28 (29%)	14/37 (38%)	0.587	0.613
Fully dependent	43/65 (66%)	18/28 (64%)	25/37 (68%)	0.023	0.881
Access to funded respite aid in place	47/65 (72%)	18/28 (64%)	29/37 (78%)	3.074	0.215
If yes, the respite received is sufficient	23/44 (52%)	8/19 (42%)	15/25 (60%)	1.417	0.492
Child/adult’s illness has affected parent’s health and wellbeing	65/66 (99%)	28/28 (100%)	37/38 (97%)	1.480	0.477
Child/adult’s illness has affected parent’s job or career	60/66 (91%)	26/28 (93%)	34/38 (89%)	0.223	0.637

### Sleep disorders and intervention

The SDSC was returned by 91% of carers (62/68), of which 71% (44/62) reported either at least one abnormal sleep subcategory or had an abnormal total sleep score (Supplementary Fig. 1). The most common sleep disorder observed was DIMS at 40% (25/62), followed by DOES at 36% (22/61). Sleep disturbance of any category was observed in 57% of individuals in the younger cohort (16/28) and 82% in the older cohort (28/34). The nature of sleep disturbance experienced also differed according to the age of individuals. The commonest sleep problem for the younger group was DIMS at 36% (10/28) compared to DOES at 52% (17/33) in the older group (Supplementary Table 3). Of those who had an abnormal sleep score in any category, 45% (20/44) received treatment through either medication or sleep hygiene and 65% found this to be successful (13/20). Parents of individuals reporting an abnormal DIMS score co-slept in 61% of cases (14/23), compared to 28% in those with a normal score (10/36), χ^2^(1) = 6.37, *P* = 0.012. Polypharmacy or single use of anti-seizure medications, including sodium valproate, topiramate, stiripentol and clobazam, was not associated with DOES.

### Predictors of long-term developmental outcome

Long-term predictors of developmental outcome/adaptive functioning were identified by using the ABAS-3 GAC score as the dependent variable. One individual harbouring a severe protein truncating variant but a near average GAC score of 77 was identified as an outlier and excluded from the prediction analyses.

A worse baseline language ability as defined by ELDQOL predicted a worse GAC score 10 years later (*P* < 0.001). A worse epilepsy severity score (*P* = 0.003) equally predicted a lower GAC score (Table 5). A high *SCN1A* genetic score similarly predicted a lower GAC score (*P* = 0.027), in particular those with an *SCN1A* genetic score > 110 (*P* = 0.011) (Table 5). Subgroup analysis revealed that in the younger group, an earlier appearance of myoclonus predicted a worse GAC score (*P* = 0.027). Usage of sodium channel blockers at any point in the 10-year follow-up period trended towards significance for predicting a worse GAC score but only for the older group (*P* = 0.092).

**Table 5:** Univariate linear regression analysis for variables predicting worse long-term developmental outcome, adjusted for age at assessment (*n* = 61)

Predictor variable (univariate)	*B*	Adjusted *R*^2^	*F*-statistic	*P*-value
All ages (*n* = 61)				
Child’s language ability	−1.64	0.204	8.54	<0.001
Epilepsy severity	2.15	0.119	4.98	0.003
*SCN1A* genetic score (linear)	−0.019	0.087	3.49	0.027
*SCN1A* genetic score > 110 (binary)	−3.38	0.116	4.42	0.011

*B*, unstandardized coefficient, age at time of diagnosis was held constant in each univariate analysis.

## Discussion

We prospectively evaluated clinical and demographic features in individuals with *SCN1A* positive Dravet syndrome over a 10-year follow-up period identifying predictors of developmental outcome and potential disease biomarkers. Our study emphasizes the very limited or absent developmental progress in adaptive behaviour and the increasing prevalence of comorbidities, including autistic features, behavioural and mobility problems at follow-up.

Caregivers reported an overall improvement in epilepsy severity across the follow-up period. This effect was more pronounced for the older age group, corroborating previous reports that seizure frequency decreases with age.^13^ Across the follow-up period, four out of seven individuals died due to SUDEP, in-keeping with previously reported studies on its role in premature mortality.^22^ SUDEP can cause significant parental anxiety and we noted that 40% of carers co-slept, likely as a preventative measure. Surprisingly, 35% of carers reported never having discussed SUDEP. Whilst these discussions might have taken place at diagnosis, carers may not recall this across the 10-year period, emphasizing the importance of having repeated discussions about risks with caregivers and how to manage them.

Half of all individuals reported dental or eating problems and 18% required supplementation with additional calories through a gastrostomy. A significant proportion (40%) of individuals experienced fractures and 10% reported to have a scoliosis. An increased risk of fractures in our cohort may be due to falls following a seizure or due to the use of drugs associated with decreased bone density in childhood, including sodium valproate.^23^ Individuals with DS should have better access to additional therapies including dieticians and physiotherapists, which less than half of carers in our cohort reported having access to.^17^

Caregivers in our cohort reaffirmed the long-term burden of illness, with 63% of individuals being fully dependent on their carers and 91% still living with their family, many of whom have reached adulthood. In a prospective multicentre study, Strzelczyk *et al*.^24^ found that 45% of DS caregivers had depressive symptoms and that management of DS was far more resource intensive than other epilepsy individuals. A recent report highlighted the range of stringent measures employed by caregivers to prevent seizures, including increasing hand hygiene to prevent infection passing onto the child.^25^ This is in contrast to other chronic diseases such as diabetes or asthma with less caregiver burden of illness.^26^

Overall, our study corroborates the poor long-term developmental outcomes for individuals with DS,^15,27^ with 51% of individuals (31/61) receiving a classification of profound cognitive impairment. This decline across the 10-year follow-up period was greater in the younger group and reflects the rapid disease progression in the first five years of life, contrasting the relative plateauing of functioning in the older group. However, this observation might be accentuated by a floor effect noted particularly among older individuals with lower ABAS-3 scores.

With this disease course in mind, we investigated whether predictors of developmental outcome at baseline continued to be significant in the same individuals 10 years later.

Several independent predictors of long-term developmental outcome were identified. Worse epilepsy severity at the time of baseline assessment predicted a lower developmental outcome at follow-up for the entire cohort, suggesting that the severity of epilepsy early on in the disease course continued to have long-term effects on cognition across the 10-year follow-up period.^28^ We observed that usage of sodium channel blockers at any point in the 10-year follow-up period trended towards being predictive of a lower GAC score in older children. Recent studies have shown usage of contraindicated medications to worsen cognition and quality of life.^21,29^

Continuity in linguistic development has an important role in cognitive development, particularly in the first five years of life.^30^ Our study shows baseline language ability as a predictor for a lower GAC score 10 years later for both age groups. An early appearance of myoclonus in younger children at baseline assessment continued to predict a worse developmental outcome 10 years later, in-keeping with the original findings from the same cohort in 2010^12^ and corroborates reports that early myoclonus has a negative prognostic impact.^31^ It remains to be seen whether newer precision therapies are able to modify the severity of comorbidities in addition to improving seizure control.

The *SCN1A* genetic score reflects variant characteristics such as paralog conservation of the mutated amino acid position and physicochemical properties (Grantham score) of the observed substitution.^20^ Our finding that a higher *SCN1A* genetic score predicted a lower GAC outcome at 10 years follow-up establishes the *SCN1A* genetic score as a potential biomarker of disease outcome. This emphasizes the role of the underlying mutation and channelopathy in the long-term developmental outcome of DS individuals.

Nearly three-quarters of individuals in this cohort were reported to experience sleep disturbance, which is greater than reported in general epilepsy cohorts^32^ and closely matches a study by Licheni *et al*.^33^ using the same SDSC on a DS cohort. The high frequency of sleep disorders in DS individuals can be attributed to many factors. A drug-naïve *SCN1A* DS mouse model demonstrated impaired sleep secondary to loss of the encoded Nav1.1 in forebrain GABAergic interneurons.^34^ However, some individuals reported a normal sleep profile in our cohort of exclusively *SCN1A* mutation-positive DS, suggesting that whilst the underlying *SCN1A* variant contributes to sleep disturbance, it is not the sole determinant. DIMS was the commonest sleep disturbance overall and may be related to the high frequency of nocturnal seizures observed in DS.^35^ Environmental factors such as co-sleeping that reduces quality of sleep may also impact DIMS. Anti-seizure medications, including sodium valproate, topiramate, stiripentol and clobazam, have been reported to cause sleep disturbance, especially DOES.^33,35^ However, we observe comparable rates of individuals using these medications in those with and without sleep disturbances. Good quality of sleep in DS remains an unmet clinical need and offering professional sleep advice may improve quality of life.

Our study has several limitations. Whilst we achieved a response rate of 60%, a significant number of individuals did not respond, leaving a smaller sample size for subgroup analysis. However, we did not identify any significant difference in demographic features comparing baseline with follow-up cohorts. Baseline cognitive data were obtained from professionals with expertise in assessing developmental outcome in DS rather than by standardized questionnaires (ABAS-3) that were used as part of the follow-up study design.

## Conclusion

This study reaffirms the poor long-term cognitive outcomes in DS and the substantial caregiver burden of illness. The negative impact of epilepsy severity at baseline on long-term developmental outcomes suggests the importance of implementing early and focused therapeutic strategies. However, few individuals in our cohort were treated with newer anti-seizure medications at baseline and the potential impact of newer agents requires further study. Our data highlight the importance of addressing the associated comorbidities and ultimately the underlying *SCN1A* channelopathy to improve quality of life for affected individuals and their carers/families.

## Supplementary Material

fcae004_Supplementary_Data

## Data Availability

The data that support the findings of this study are available from the corresponding author, upon reasonable request.
