# Supplementary material for: Long-term predictors of developmental outcome and disease burden in SCN1A-positive Dravet syndrome
Source: Brain Commun. 2024 Jan 9;6(1):fcae004. doi: 10.1093/braincomms/fcae004 (PMC10789590; doi:10.1093/braincomms/fcae004)
Supplement: fcae004_Supplementary_Data [file fcae004_supplementary_data.pdf]

Supplementary Tables

**Supplementary Table 1.** Demographic and phenotypic cohort description								
	**All Ages (68)**		**Younger Cohort (28)**		**Older Cohort (40)**		**Chi-square test**	
**Feature**	**Median (IQR)**	**Occurrence number/total (%)**	**Median (IQR)**	**Occurrence number/total (%)**	**Median (IQR)**	**Occurrence number/total (%)**	**X^2**	***p*-value**
Age (years)*	17 (14 – 24)	68/68 (100%)	13 (12 – 14)	68/68 (100%)	23 (19 – 26)	68/68 (100%)	-	-
Proportion of cohort male†		36/68 (53%)		10/28 (36%)		26/40 (65%)	5.670	0.017
Proportion of mutations (truncating / truncating + non-truncating)†		41/68 (60%)		19/28 (68%)		22/40 (55%)	1.137	0.286
First seizure (age, months)†	5 (4 – 7)	64/68 (94%)	6 (4 – 7)	27/28 (96%)	5 (4 – 7)	37/40 (93%)	0.459	0.498
Hemiclonic seizure (age, months)†	7 (5 – 9)	39/68 (57%)	6.5 (5 – 10)	16/28 (57%)	7 (5 – 9)	23/40 (58%)	<0.001	0.976
Generalised tonic-clonic seizures (age, months)†	8 (5 – 12)	53/68 (78%)	7 (5 – 11)	25/28 (89%)	8.5 (5 – 12)	28/40 (70%)	3.563	0.059
Status Epilepticus (age, months)†	6 (4 – 8)	28/68 (41%)	6 (4 – 7)	11/28 (39%)	6 (5 – 9)	17/40 (43%)	0.073	0.791
Focal seizures with impairment of awareness (age, months)†	16 (6 – 36)	22/68 (32%)	17 (6 – 36)	9/28 (32%)	15 (6 – 42)	13/40 (33%)	0.001	0.975
Myoclonic seizures (age, months)†	14 (10 – 24)	47/68 (69%)	15 (9 – 24)	18/28 (64%)	13 (12 – 25)	29/40 (73%)	0.521	0.471
Atypical absence (age, months)†	14.5 (11 – 26)	28/68 (41%)	13 (13 – 25)	10/28 (36%)	20 (12 – 30)	18/40 (45%)	0.586	0.443
Abnormal Interictal EEG in first 6 months (y/n)†		1/32 (3%)		1/15 (7%)		0/17	-	-
Abnormal Interictal EEG in first 7-12 months (y/n)†		13/29 (45%)		7/16 (44%)		6/13 (46%)	0.015	0.695
Abnormal Interictal EEG in first 13-24 months (y/n)†		8/14 (57%)		4/6 (67%)		4/8 (50%)	0.389	0.532

First Seizure Precipitated By:†<br>Fever/Illness<br>No precipitant<br>Bath<br>Vaccination		41/65 (63%)<br>20/65 (31%)<br>0/65<br>4/65 (6%)		19/26 (73%)<br>7/26 (27%)<br>0/26<br>0/26		22/39 (56%)<br>13/39 (33%)<br>0/39<br>4/39 (10%)	1.860<br>0.309<br>-<br>-	0.173<br>0.583<br>-<br>-
Photosensitivity (y/n)†		13/61 (21%)		2/26 (8%)		11/35 (31%)	3.697	0.054
MRI abnormality (y/n)†		5/58 (9%)		2/25 (8%)		3/33 (9%)	0.0215	0.883

*at follow-up, †at baseline

**Supplementary Table 2**. Medications indicated by caregivers to exacerbate seizure frequency	
**Medications increasing seizure frequency**	**Occurrence number / total (%), follow-up**
Lamotrigine	23/47 (49%)
Carbamazepine	11/47 (23%)
Phenytoin	7/47 (15%)
Levetiracetam	6/47 (13%)
Valproate	3/47 (6%)

**Supplementary Table 3.** Frequency of children with abnormal total and subcategory sleep scores (n = 62)			
**Abnormal Sleep Category**	**Complete Cohort**	**0-5 -years-old**	**≥ 6-years-old**
DIMS	25/62 (40%)	10/28 (36%)	15/34 (44%)
SBD	17/62 (27%)	7/28 (25%)	10/34 (29%)
DA	5/61 (8%)	1/28 (4%)	4/33 (12%)
SWTD	21/62 (34%)	9/28 (32%)	12/34 (35%)
DOES	22/61 (36%)	5/28 (18%)	17/33 (52%)
SHY	3/62 (5%)	1/28 (4%)	2/34 (6%)
Total Sleep Score	23/58 (40%)	10/28 (36%)	13/30 (43%)
Any Category	44/62 (71%)	16/28 (57%)	28/34 (82%)
Disorders of initiating and maintaining sleep (DIMS), sleep breathing disorders (SBD), disorders of arousal (DA), sleep-wake transition disorders (SWTD), disorders of excessive somnolence (DOES) and sleep hyperhidrosis (SH).			

Supplementary Figure

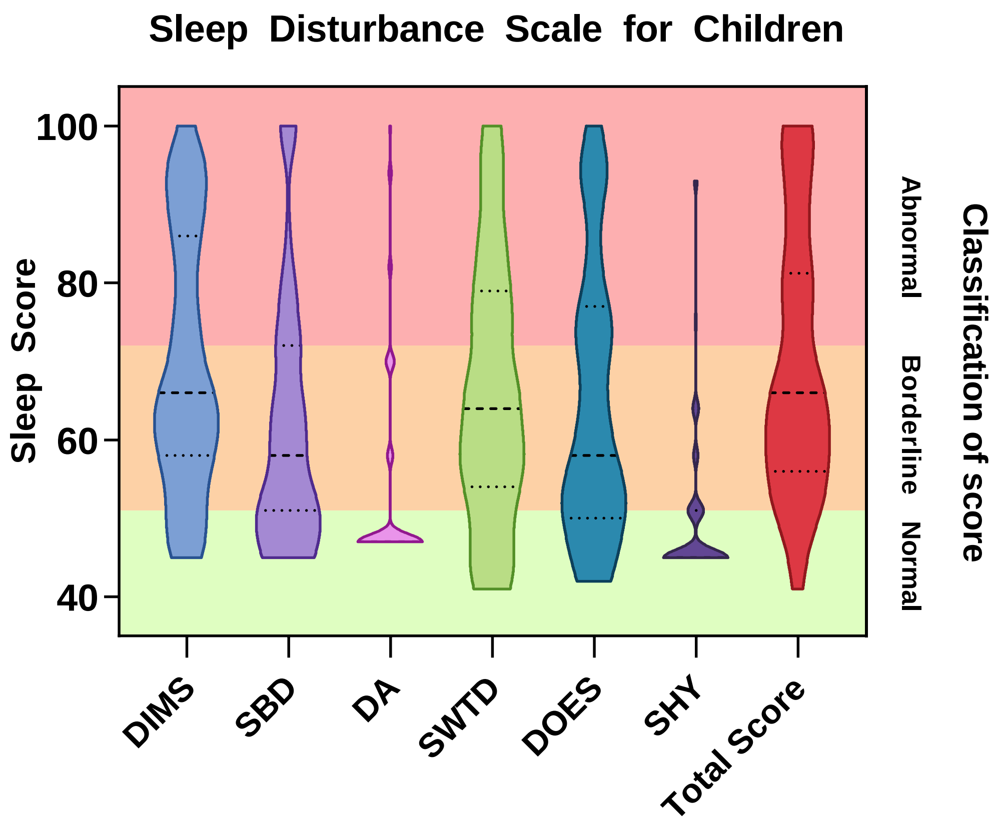

Supplementary Figure 1. Violin plot of T-score and pathological classification for patients across different sleep categories. The bold dashed line represents the median and the dotted line is the interquartile range. DIMS, disorders of initiating or maintaining sleep; SBD, sleep breathing disorders; DA, disorders of arousal; SWTD, sleep–wake transition disorders; DOES, disorders of excessive somnolence; SHY, sleep hyperhidrosis.
